# Characterization of coagulase negative staphylococci from cases of subclinical mastitis in dairy cattle in Kampala, Uganda

**DOI:** 10.1186/2046-0481-67-12

**Published:** 2014-06-02

**Authors:** Sandra Björk, Renee Båge, Benon M Kanyima, Susanne André, Maria G Nassuna-Musoke, David O Owiny, Ylva Persson

**Affiliations:** 1 Division of Reproduction, Department of Clinical Sciences, Swedish University of Agricultural Sciences (SLU), Uppsala, Sweden; 2 College of Veterinary Medicine, Animal Resources and Biosecurity, Makerere University, Kampala, Uganda; 3 Department of Bacteriology, National Veterinary Institute (SVA), Uppsala, Sweden; 4 Department of Animal Health and Antimicrobial Strategies, National Veterinary Institute/Växa Sverige, Uppsala, Sweden

**Keywords:** CNS, *Staphylococcus epidermidis*, *Staphylococcus haemolyticus*, MALDI-TOF

## Abstract

**Background:**

Coagulase negative staphylococci (CNS) are the most common pathogens leading to subclinical mastitis (SCM) in dairy cattle in Uganda. Coagulase negative staphylococci can vary between bacterial species in how they cause disease. The aim of the study was to characterize CNS, from cows with SCM in Uganda, at the species level.

**Findings:**

Quarter milk samples (n = 166) were collected from 78 animals with SCM. Bacteriological analyses were carried out at Makerere University, Kampala, Uganda and at the National Veterinary Institute (SVA), Uppsala, Sweden. The most common pathogens found in milk samples from cows with SCM were CNS (31.7%). Two species of CNS were found, *S. epidermidis* (85%) and *S. haemolyticus* (15%). Of the CNS isolates, 16/20 (80%) were positive for β-lactamase production (β+).

**Conclusions:**

In milk samples from cows with SCM caused by CNS, *S. epidermidis* was most prevalent, followed by *S. haemolyticus*.

## Findings

Subclinical mastitis is common in dairy cows in Uganda [1,2]. Previous studies [1-3] have found coagulase negative staphylococci (CNS) to be the most common pathogens in subclinical mastitis (SCM) in dairy cattle in Uganda. Different species of CNS can have different properties (eg. chronicity, which cows they infect, if they cause clinical or subclinical mastitis etc.) and the prevalence can also vary between countries and regions [4-7]. Characterization of CNS, from cases of mastitis, at the species level has never previously been performed in Uganda. The aim of this study was therefore to characterize different species of CNS, from milk samples collected from urban and peri-urban dairy cows with SCM in the Kampala region in Uganda.

The CNS isolates were cultured from quarter milk samples from cows with SCM, collected in a previous study [3,8]. In brief, quarter milk samples (n = 166) were aseptically collected from Holstein Friesian crossbred cows (n = 78) in 17 dairy farms in and around Kampala, Uganda. The herd size varied from 1 to 67 lactating cows (mean value 19.5, median value 12). All cows were hand milked twice daily except on one farm where cows were milked three times a day. Cows were managed either in zero- or open-grazing systems. No data on somatic cell counts were available.

Milk from cows with no symptoms from the udder or visible changes in milk were examined by California Mastitis Test (CMT) at afternoon milking and the diagnosis was solely based on a CMT value ≥ 3 in quarter milk (using the Scandinavian scoring from 1 to 5, where 1 is negative and 5 is representing the highest SCC). Bacteriological examination of SCM cases was limited to samples with CMT ≥ 4 (166 milk samples collected from 78 cows).

Bacteriological analyses were done at Makerere University, Kampala, Uganda, and at the accredited mastitis laboratory at the National Veterinary Institute (SVA), Uppsala, Sweden.

Quarter milk samples were examined for bacterial growth by collecting 10 μl of the milk with a sterile loop and spreading it on 5% bovine blood agar plates with aesculine. Plates were evaluated at 24 hours and 48 hours after aerobic incubation at 37°C. A preliminary identification of the species of bacteria growing was based on bacterial colony characteristics [9]. Growth of ≥ 3 colony-forming units (CFUs) was considered as positive growth. A growth of ≥ 2 different microorganisms was classified as mixed flora. The potassium hydroxide test was made to distinguish between Gram positive and Gram negative bacteria and the catalase test (hydroperoxide) was used to separate streptococci from staphylococci. For all isolates of streptococci, staphylococci and uncertain isolates, 1 μ of bacterial isolate was inoculated with a loop into cryo tubes containing blood agar base no 2, oxoid CM0271 and 5% horse serum for transportation in ice pack-cooler box to SVA, Sweden, for further analyses.

Staphylococcal isolates were analyzed using the reversed CAMP-reaction test and the coagulase test. In addition, Matrix Assisted Laser Desorption Ionization-Time of Flight mass spectrometry (MALDI-TOF) was performed on CNS isolates.

All CNS isolates were examined for β-lactamase production by the “clover-leaf” method as described by [10].

Coagulase negative staphylococci were the most common pathogens, found in 31.7% (followed by *Streptococcus agalactiae* (22.2%)) of isolates with growth of specific mastitis pathogens (i.e. mixed flora excluded). Of these, two species of CNS were found, *S. epidermidis* (85%) and *S. haemolyticus* (15%). Of all CNS isolates, 16/20 (80%) were positive for β-lactamase production (β+); a majority (15/17, 88%) of the *S. epidermidis* isolates and one out of three (33%) of the *S. haemolyticus* isolates.

For SCM, other studies from Uganda [1,2] also reported CNS as the most common pathogens. Due to practical reasons, only subclinical cases with a CMT-score of ≥ 4 were sampled for bacteriological culture in this study, and therefore the bacteriological results do not reflect the definition of SCM used in the original study (CMT ≥ 3) [3].

In our study, *S. epidermidis* was most prevalent, followed by *S. haemolyticus*. There are few studies from Africa on different CNS species, but in a study from Zimbabwe on both clinical mastitis and SCM, the most frequently isolated species were *S. chromogenes*, *S. epidermidis* and *S. hominis*[11]. A Swedish study on different CNS, [12] reported that *S. epidermidis* was the most common CNS in SCM and that *S. haemolyticus* was also quite common. A Belgian study [13] reported *S. chromogenes* and *S. haemolyticus* to be the most common species in milk samples from cows with intramammary infections (IMI). Moreover, for *S. haemolyticus*, the environment was found as a reservoir, suggesting that IMI with these species were possibly environmental in origin [13,14]. *Staphylococcus epidermidis* is described as a more udder-adapted pathogen [13,14]. Persistent IMI were common in quarters infected with *S. epidermidis* indicating a more udder-adapted origin [6]. There was also a close genotypic relationship between *S. epidermidis* isolated from different cows on the same farm suggesting clonal dissemination [15]. Yet other studies, [15,16] suggest a human source for *S. epidermidis* udder infections, which is supported by the fact that all cows in this study are hand milked, and that *S. epidermidis* is associated with suboptimal milking hygiene, facilitating the spread of *S. epidermidis* from cow to cow. Most other studies on different CNS species have found more species (eg. 14 species in [12]), while this study only found two CNS species. The reason for this is not clear, but the number of cows and herds is small and from the same region so the results may not reflect the entire Ugandan situation.

Eighty percent of isolates from SCM were β + in this study. These figures are similar to what was found in other studies on staphylococci causing mastitis in Uganda [1,17]. In a study from Sweden, the corresponding figure was 38% [18]. Among the most frequently isolated CNS species in Sweden, β-lactamase production was more common in *S. epidermidis* and *S. haemolyticus*[12]. The lower frequency of β + isolates in Sweden compared to Uganda is probably due to better infectious disease control, where the spread of betalactamase producing staphylococci is prevented and also partly due to more prudent use of antibiotics. Observations, although risk factors were not included in this study, during data collection in Uganda implied a wide use of broad-spectrum antibiotics during lactation for treating mastitis as well as poor hygiene during milking and sub optimal milking technique and routines (eg. strip milking, no milking order etc.). This was the first study on different CNS species in Uganda. The number of cows include were limited and specific risk factors were not evaluated. Further studies would be necessary to achieve this.

## Competing interests

The authors declare that they have no competing interests.

## Authors’ contributions

SB carried out the field study, performed the statistics and drafted the manuscript. RB participated in the design of the study and helped to draft the manuscript, BK participated in the field study and in the design of the study and helped to draft the manuscript, SA performed the laboratory work at SVA and helped to draft the manuscript, MN and DO participated in the design of the study and helped to draft the manuscript, YP conceived of the study, and participated in its design and coordination and helped to draft the manuscript. All authors read and approved the final manuscript.
